# Elevated IL-22 in psoriasis plays an anti-apoptotic role in keratinocytes through mediating Bcl-xL/Bax

**DOI:** 10.1007/s10495-020-01623-3

**Published:** 2020-07-06

**Authors:** Bo Wang, Dan Han, Fei Li, Weikun Hou, Lijuan Wang, Liesu Meng, Kuanhou Mou, Shemin Lu, Wenhua Zhu, Yan Zhou

**Affiliations:** 1 Department of Dermatology, The First Affiliated Hospital of Xi’an Jiaotong University, Xi’an, Shaanxi China; 2 Center for Translational Medicine, The First Affiliated Hospital of Xi’an Jiaotong University, Xi’an, Shaanxi China; 3 Department of Biochemistry and Molecular Biology, School of Basic Medical Sciences, Xi’an Jiaotong University Health Science Center, Xi’an, Shaanxi China; 4 Osteonecrosis and Joint Reconstruction Ward, Joint Surgery, Xi’an Honghui Hospital, Xi’an Jiaotong University Health Science Center, Xi’an, Shaanxi China

**Keywords:** IL-22, Psoriasis, Anti-apoptosis, Keratinocytes, Bcl-xL/Bax

## Abstract

IL-22 is known to mediate inflammation in psoriasis, while IL-22 binding protein (IL-22BP) binds IL-22 to suppress IL-22 signaling. However, the function of IL-22 in regulating apoptosis in psoriasis remains poorly understood. In this study, we found that IL-22/IL-22R1 in lesional skin and IL-22 in serum from psoriatic patients were highly upregulated compared with healthy controls, while IL-22BP was not changed. Correlations between IL-22/IL-22R1 levels and the thickness of psoriatic lesions suggested that IL-22 might positively regulate abnormal hyperplasia in psoriasis. Apoptotic keratinocytes were increased only in stratum corneum, but not in spinous and basal layers of psoriasis. Moreover, IL-22 promoted cell viability in human epidermal keratinocytes (HEKs). The apoptosis induced by TNF-α and IFN-γ was inhibited in HEKs treated with IL-22, since that IL-22 upregulated Bcl-xL and downregulated Bax production in HEKs in the presence of TNF-α and IFN-γ. In addition, IL-22BP could counteract the anti-apoptotic effect of IL-22. Our finding demonstrates that IL-22 might play an anti-apoptosis role on keratinocytes to balance cell proliferation and apoptosis in psoriatic epidermis.

## Introduction

Psoriasis is widely regarded as a chronic autoimmune disease with an incidence of approximately 1% to 10% in the world [1, 2], characterized by protuberant clinical features of scaly patches on the skin. Prominent pathological features are keratinocyte hyperproliferation, incompletely differentiated epidermal keratinocytes, and abnormal keratinocyte apoptosis, which might be one of the key pathogenetic mechanisms in psoriasis [3].

In recent years, Th22 cells were proposed as a new helper T cell subset, and subsequent studies found that Th22 cells were involved in the pathogenesis of various autoimmune diseases, including psoriasis [4]. IL-22 is the representative cytokine of Th22 cells, whose membrane-bound IL-22 receptor 1 (IL-22R1) is crucial for maintaining cutaneous epithelial integrity, and its malfunction mediates abnormal epidermal proliferation [5]. IL-22 binds to the IL-22R complex to induce a cascade of downstream signaling pathways [6]. Studies have shown that pathways of IL-22 are mainly activated by signals such as STAT3 in intestinal epithelial cells [7]. IL-22 can promote IL-23-induced skin acanthosis by activating STAT3, and keratinocytes are one of the main target cells of IL-22 [8]. Additionally, IL-22 binding protein (IL-22BP), a soluble receptor for IL-22 and expressing in monocytes, activated T cells and epithelial cells, binds IL-22 to suppress IL-22 signaling [9], whose affinity to IL-22 is significantly higher than that of IL-22R1. However, IL-22BP in the treatment of psoriasis is still poorly understood until now.

The Bcl-2 family is the most important family of apoptosis regulators, including anti-apoptotic proteins (Bcl-xL, Bcl-2) and pro-apoptotic proteins such as Bax and Bak [10]. Downregulation of Bcl-xL and upregulation of Bax in lesional psoriatic skin after infliximab therapy demonstrates the contribution of Bcl-xL/Bax to the development of psoriasis [11]. IL-22 treatment in a liver injury model significantly upregulated the expression of STAT3 and Bcl-xL indicating its anti-apoptotic effects in liver damage [12]. However, the role of IL-22 in the apoptosis of psoriasis has not been reported.

In this study, we have explored the relation between IL-22 levels and the severity of psoriasis, and the role of IL-22 in mediating keratinocytes apoptosis in keratinocytes. Our results reveal an anti-apoptotic effect of elevated IL-22 in psoriasis on keratinocytes by regulating Bcl-xL/Bax.

## Results

### IL-22/IL-22R1 expression was increased in psoriasis with abnormal apoptotic keratinocytes

**Fig. 1:**
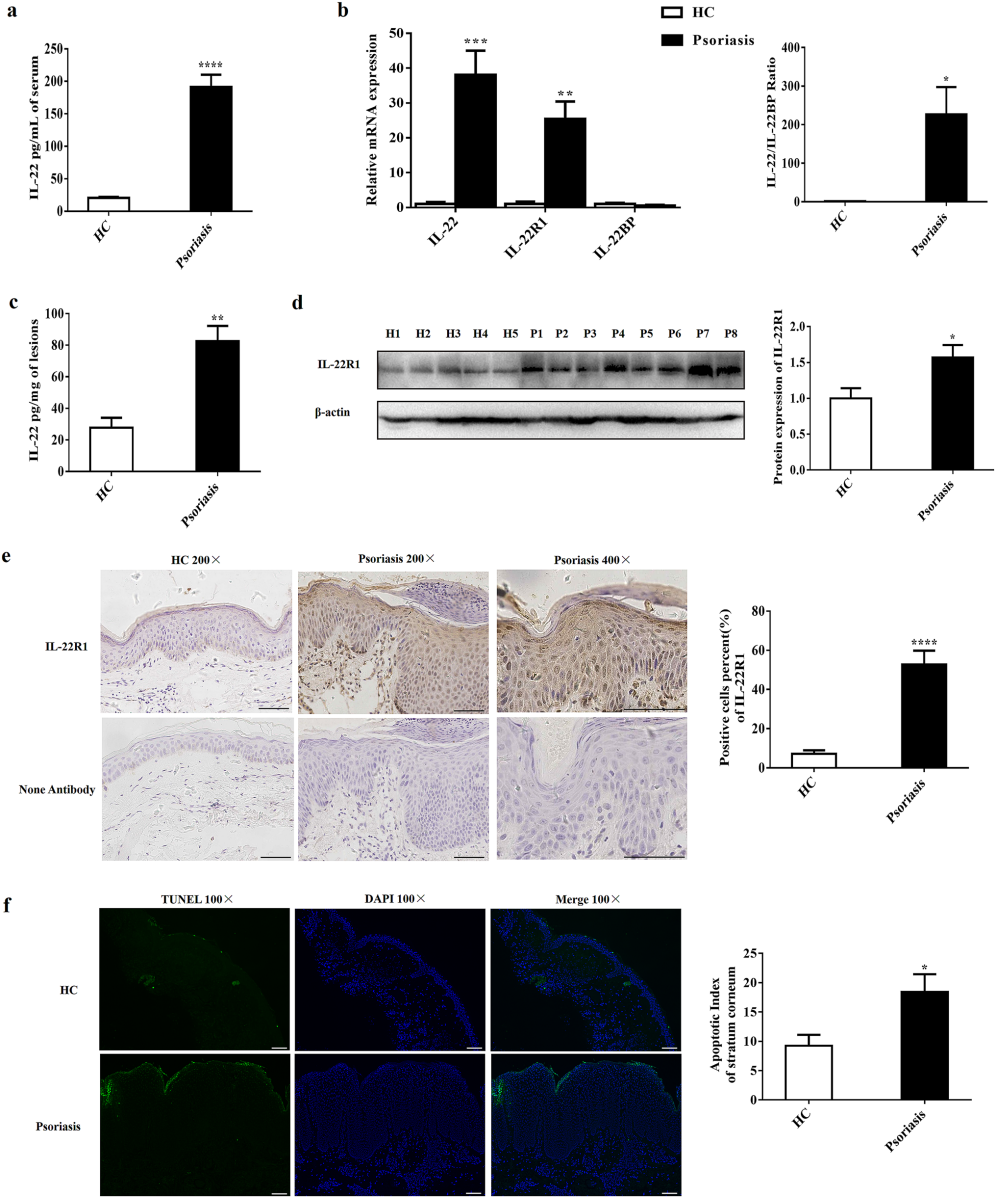
Increased expression levels of IL-22/IL-22R1 in psoriasis with abnormal apoptotic keratinocytes. **a** ELISA analysis of IL-22 expression of serum from patients with psoriasis (n = 53) and HC (n = 30). **b** RT-qPCR analysis of IL-22, IL-22R1, IL-22BP and the ratio of IL-22 to IL-22BP mRNA expression in lesional skin from patients with psoriasis (n = 23) and HC (n = 12). **c** ELISA analysis of IL-22 expression of lesions from patients with psoriasis (n = 8) and normal skin from HC (n = 5). **d** The expression of protein were detected by Western blotting and compared in psoriasis (n = 8) and normal skin from HC (n = 5). The expression of IL-22R1 was determined by immunohistochemical staining (**e**) and apoptotic keratinocytes were detected by TUNEL technique (**f**) in lesional skin from psoriasis patients and normal skin (Scale bar = 100 μm), and the percentages of IL-22R1-positive cells (**e**) and apoptotic index (**f**) in stratum corneum from psoriasis (n = 23) and HC (n = 12) were shown. Results of mRNA are normalized to β-actin expression. (*P < 0.05, **P < 0.01, ***P < 0.001, ****P < 0.0001, *t* test)

To investigate the role of IL-22, we first studied its expression regulation in psoriasis. Here we found that the concentration of IL-22 in serum was significantly higher in psoriasis patients than that in healthy controls (HC) (191.1 ± 19.24 pg/mL vs. 20.49 ± 1.739 pg/mL, respectively) (Fig. 1a, *P* < 0.0001), consistent with other reports [13, 14]. The mRNA expression level of IL-22 was increased by 38 times (*P* < 0.001) and IL-22R1 was increased by 25 times (*P* < 0.01) by quantitative real-time PCR (RT-qPCR) detection (Fig. 1b). No significant difference in IL-22BP expression in the lesions of psoriasis was observed compared with HC (Fig. 1b, *P* > 0.05). In addition, the mRNA expression of the IL-22/IL-22BP ratio increased 227-fold in lesions of psoriasis vs. HC (Fig. 1b, *P* < 0.05). In line with mRNA expression, the protein expression level of IL-22 was higher in lesions from psoriasis patients than that in HC by ELISA detection (Fig. 1c, *P* < 0.01), and IL-22R1 was increased significantly (*P* < 0.05) by Western blotting detection (Fig. 1d).

Histologically, the percentage of positive-stained cells of IL-22R1 (*P* < 0.0001) was increased in epidermis from psoriasis vs. HC (Fig. 1e). The apoptotic features in psoriasis are consistent with the findings of Kristofer Thorslund et al. [15]. The terminal deoxynucleotidyl transferase dUTP nick-end labeling (TUNEL) staining showed that the apoptotic keratinocytes, mainly located in stratum corneum of psoriasis, were increased compared with HC (*P* < 0.05) (Fig. 1f). However, up-regulation of strong stained membrane IL-22R1 was mainly observed in spinous and basal layers, where few TUNEL positive cells were observed (Fig. 1e, f). Our data suggested that elevated IL-22 probably mediated the balance between cell proliferation and apoptosis in psoriatic epidermis.

### The expression of IL-22/IL-22R1 was positively correlated with the severity of psoriatic lesions

**Fig. 2:**
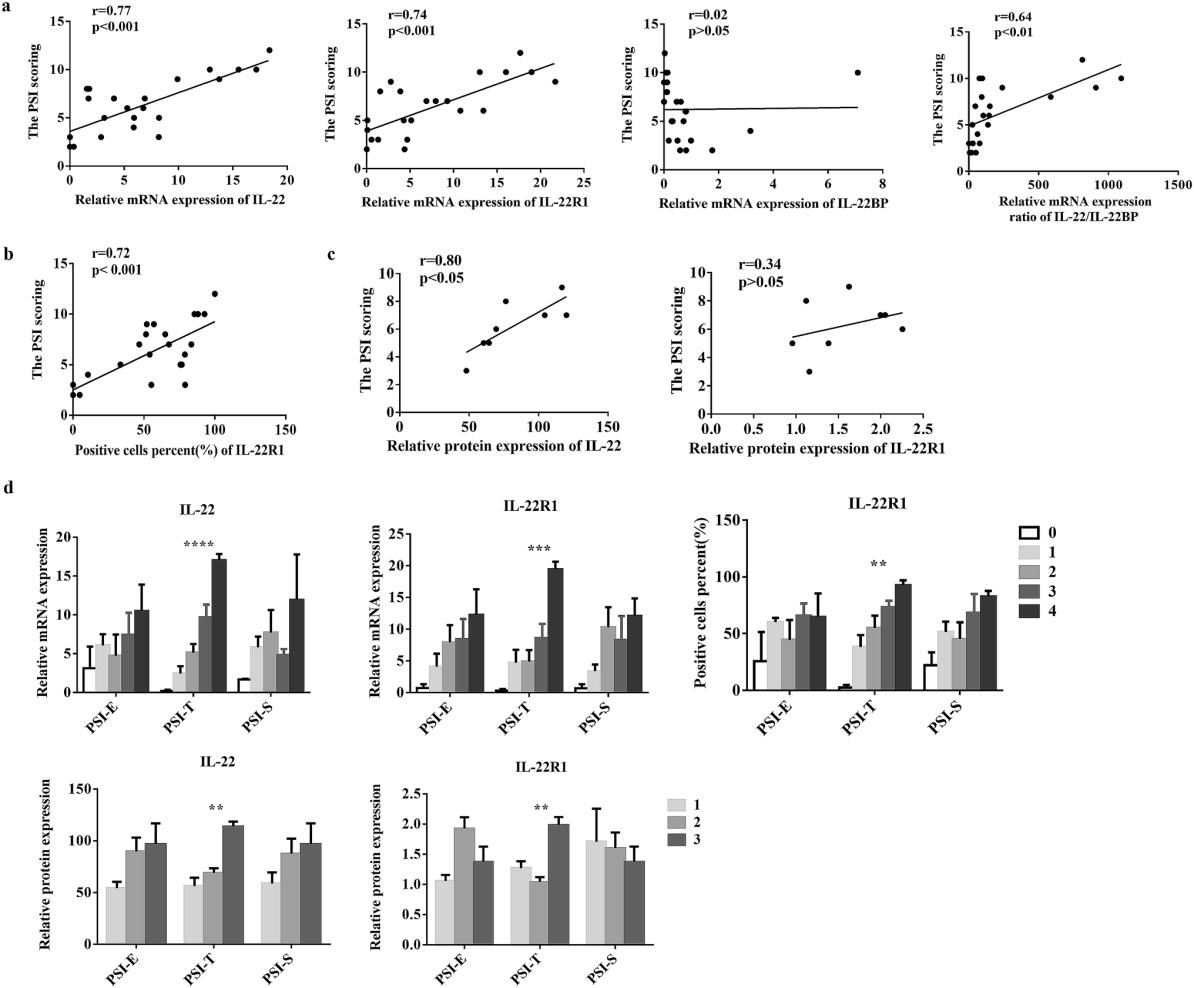
Positive correlations between the expression of IL-22/IL-22R1 and severity in lesions of psoriasis. The correlation analysis of mRNA expression level of IL-22, IL-22R1, IL-22BP and IL-22/IL-22BP ratio (n = 23) (**a**), the percentages of IL-22R1-positive cells (n = 23) (**b**), the protein expression level of IL-22, IL-22R1 (n = 8) (**c**) and the PSI scores of psoriatic lesions. **d** The difference in the mRNA (n = 23) and protein (n = 8) expression levels of IL-22/IL-22R1 and the percentages of IL-22R1-positive cells (n = 23) in skin lesions from psoriasis with different PSI-E, PSI-T and PSI-S scores. **a, b** and **c** were analyzed by Pearson correlation analysis; **d** by one-way ANOVA followed by Tukey's multiple comparisons test. (**P < 0.01, ***P < 0.001, ****P < 0.0001)

To evaluate the correlation of IL-22/IL-22R1 and clinical parameters of psoriasis patients, we analyzed the mRNA and protein levels of IL-22/IL-22R1 and psoriasis severity index (PSI) by Pearson correlation analysis. The mRNA levels of IL-22 (*r* = 0.77, *P* < 0.001), IL-22R1 (*r* = 0.74, *P* < 0.001) and the protein levels of IL-22 (*r* = 0.80, *P* < 0.05) were correlated positively with the PSI (Fig. 2a, c), while the protein levels of IL-22R1 (*r* = 0.34, *P* > 0.05) were not correlated positively with the PSI (Fig. 2c). Although no significant correlation was observed between mRNA levels of IL-22BP and the PSI (*r* = 0.02, *P* > 0.05), the ratio of IL-22 to IL-22BP (*r* = 0.64, *P* < 0.01) was still correlated positively with the PSI (Fig. 2a). In addition, the percentage of IL-22R1-positive cells (*r* = 0.72, *P* < 0.001) in epidermis of psoriasis was significantly correlated with PSI (Fig. 2b).

To accurately illustrate the correlation between IL-22/IL-22R1 and the severity of the psoriasis lesions, we further compared their expression (Fig. 2d). There was no significant difference in IL-22/IL-22R1 mRNA levels in lesions with different PSI-E (erythema) and PSI-S (scales) scores (*P* > 0.05). However, IL-22/IL-22R1 mRNA levels were different with varying PSI-T (thickness) scores. The mRNA levels of IL-22 (*P* < 0.0001) and IL-22R1(*P* < 0.001) and the percentage of IL-22R1-positive cells (*P* < 0.01) in the patients (score = 4) were higher than those of the patients (score = 0–3) (Fig. 2d). The protein levels of IL-22 and IL-22R1 in the patients (score = 3) were higher than those of the patients (score = 1, 2) (*P* < 0.01) (Fig. 2d). Altogether, these results suggested that IL-22 might participate in developing abnormal hyperplasia in psoriasis.

### The expression of apoptosis-related genes was increased in the lesions of psoriasis

**Fig. 3:**
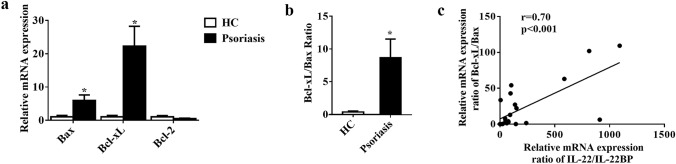
Increased expression levels of apoptotic-related genes in lesions of psoriasis. **a** RT-qPCR analysis of Bax, Bcl-xL and Bcl-2 in lesional skin from patients with psoriasis (n = 23) and normal skin from HC (n = 12). **b** The ratio of Bcl-xL to Bax mRNA expression levels in lesional skin from psoriasis (n = 23) and HC (n = 12). **c** The correlation analysis of mRNA expression levels of IL-22/IL-22BP ratio and Bcl-xL/Bax ratio of psoriatic lesions (n = 23). **a**, **b** were analyzed by *t* test; **c** by Pearson correlation analysis. (*P < 0.05)

Next, we examined the expression of apoptosis-related genes in lesions of psoriasis. The mRNA expression levels of the pro-apoptosis gene Bax (*P* < 0.05) and the anti-apoptosis gene Bcl-xL (*P* < 0.05) were significantly upregulated in psoriasis lesions (n = 23) compared with HC (n = 12) by RT-qPCR, while Bcl-2 was not changed (Fig. 3a). Compared with HC, the ratio of Bcl-xL to Bax was higher in psoriasis (*P* < 0.05) (Fig. 3b). We found that Bax was increased by 6 times, Bcl-xL was increased by 22 times, and their ratio was increased by 24 folds. These results indicated that Bcl-xL and Bax mediated anti-apoptosis and apoptosis in psoriasis. We also found a positive correlation between IL-22/IL-22BP and the ratio of Bcl-xL to Bax (*r* = 0.70, *P* < 0.001, Fig. 3c). Therefore, we proposed that IL-22 might regulate apoptosis through mediating Bcl-xL and Bax.

### IL-22 promoted cell proliferation and inhibited apoptosis in HEKs

**Fig. 4:**
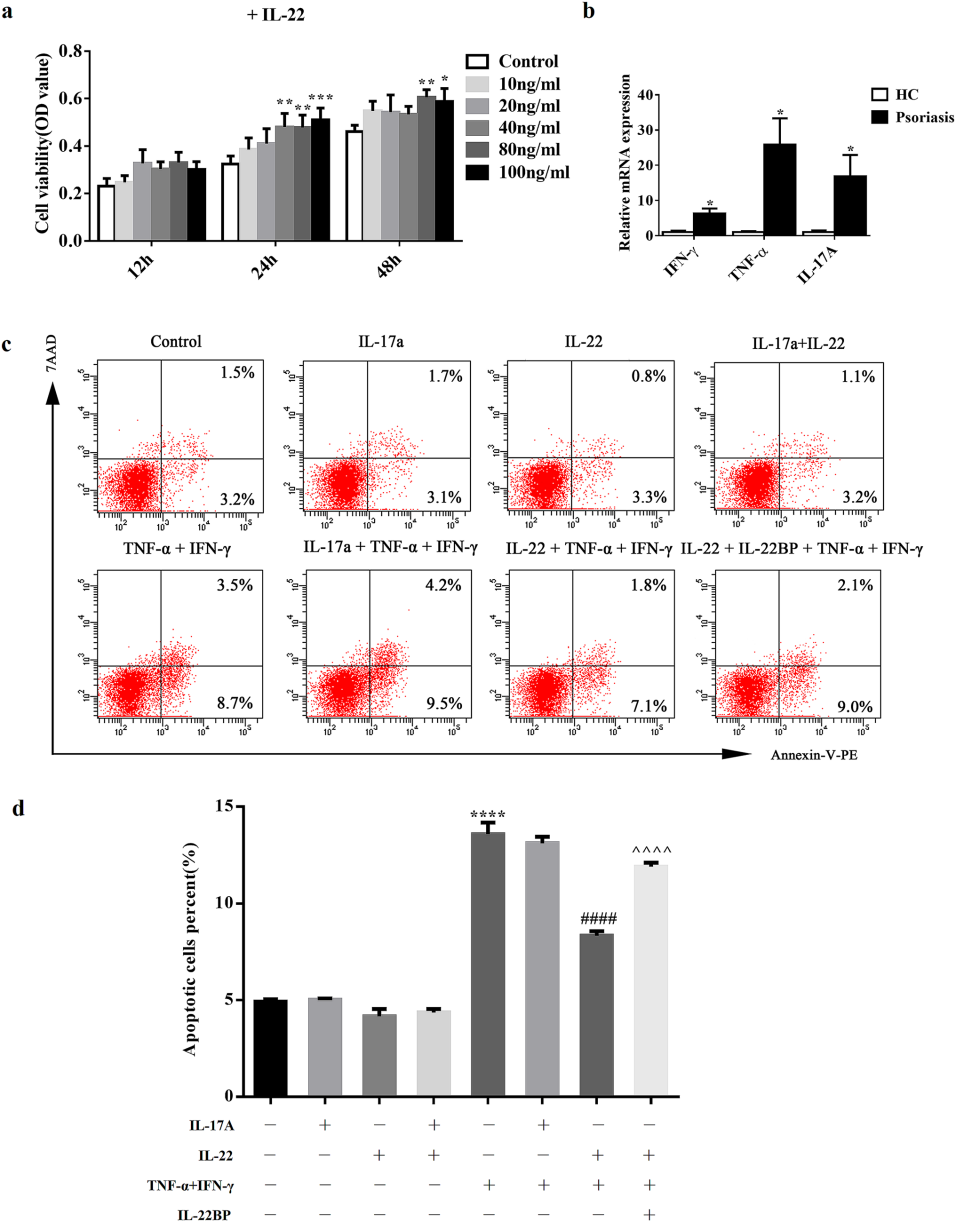
Proliferation and anti-apoptosis effects in HEKs treated with IL-22. **a** The effects of IL-22 of 10 ng/mL, 20 ng/mL, 40 ng/mL, 80 ng/mL and 100 ng/mL on the proliferation of primary keratinocytes were detected by MTT assay at 12 h, 24 h and 48 h respectively. **b** RT-qPCR analysis of TNF-α, IFN-γ and IL-17A in lesional skin from patients with psoriasis (n = 23) and normal skin from HC (n = 12). **c** Representative apoptotic rate of primary keratinocytes treated with IL-22 in different condition by FACS after double staining with 7AAD and Annexin V-PE at 24 h. **d** Statistical analysis of apoptotic rate of keratinocytes. **a** and **d** were analyzed by two-way ANOVA and one-way ANOVA respectively followed by Tukey's multiple comparisons test; **b** by *t* test. (*: vs control; #: vs TNF-α + IFN-γ; ^: vs IL-22 + TNF-α + IFN-γ. *P < 0.05, **P < 0.01, ***P < 0.001, ****P < 0.0001; ####*P* < 0.0001; ^^^^*P* < 0.0001). n ≥ 3

To further confirm the role of IL-22 in mediating keratinocyte proliferation in psoriasis, we used 10, 20, 40, 80 and 100 ng/mL of IL-22 to stimulate normal human epidermal keratinocytes (HEKs), and the proliferation of cells was detected by 3-(4,5-Dimethylthiazol-2-yl)-2,5-diphenyltetrazolium bromide (MTT) method after 12 h, 24 h and 48 h. The optical density (OD) values with IL-22 treatment (40 ng/mL: *P* < 0.01, 80 ng/mL: *P* < 0.01 and 100 ng/mL: *P* < 0.001) were increased compared with controls after 24 h. Meanwhile, the OD values were upregulated by 80 ng/mL (*P* < 0.01) and 100 ng/mL (*P* < 0.05) of IL-22 treatment after 48 h (Fig. 4a).

TNF-α and IFN-γ are well known as inducers of inflammation in psoriasis, which can also be considered as apoptosis inducers [16, 17]. As expected, TNF-α (*P* < 0.05) and IFN-γ (*P* < 0.05) were significantly elevated in the lesions of psoriasis compared with HC by RT-qPCR (Fig. 4b). IL-17A was reported to have an anti-apoptotic effect in experimental autoimmune encephalomyelitis (EAE) or under the other conditions [18, 19]. Therefore, we are also very interested in its role in apoptosis in psoriasis and found that IL-17A was dramatically increased in psoriatic lesions (n = 23) vs. HC (n = 12) by RT-qPCR (Fig. 4b) (*P* < 0.05). However, there was no correlation between IL-17A and the ratio of Bcl-xL to Bax, suggesting that IL-17A might not modulate Bcl-xL/Bax activation in psoriasis.

To disclose whether IL-22 could directly affect the apoptosis of human keratinocytes, we investigated the effects of IL-22 exposure on HEKs by flow cytometry in different conditions. As shown, IL-22 alone did not play strong effects of anti-apoptosis. However, IL-22 could inhibit apoptosis of HEKs pretreated with TNF-α and IFN-γ for 24 h (Fig. 4c). Compared with controls, the apoptotic rate was increased by 1.76 times in HEKs induced with TNF-α and IFN-γ (*P* < 0.0001). The apoptotic rate of these cells treated with IL-22 was then inhibited by 38.61% (*P* < 0.0001), and IL-22BP partially reversed the anti-apoptotic rate of IL-22 by 42.81% (*P* < 0.0001) (Fig. 4d). Interestingly, IL-17A alone or combined with IL-22/ TNF-α and IFN-γ did not affect original apoptosis of HEKs (*P* > 0.05) (Fig. 4d), indicating that IL-17A does not influence apoptosis in a pro-inflammatory environment. In summary, IL-22 inhibited the apoptosis of HEKs induced by TNF-α and IFN-γ, independently with IL-17A, which could be partly counteracted by IL-22BP.

### Bcl-xL was increased while Bax was decreased in HEKs treated with IL-22

**Fig. 5:**
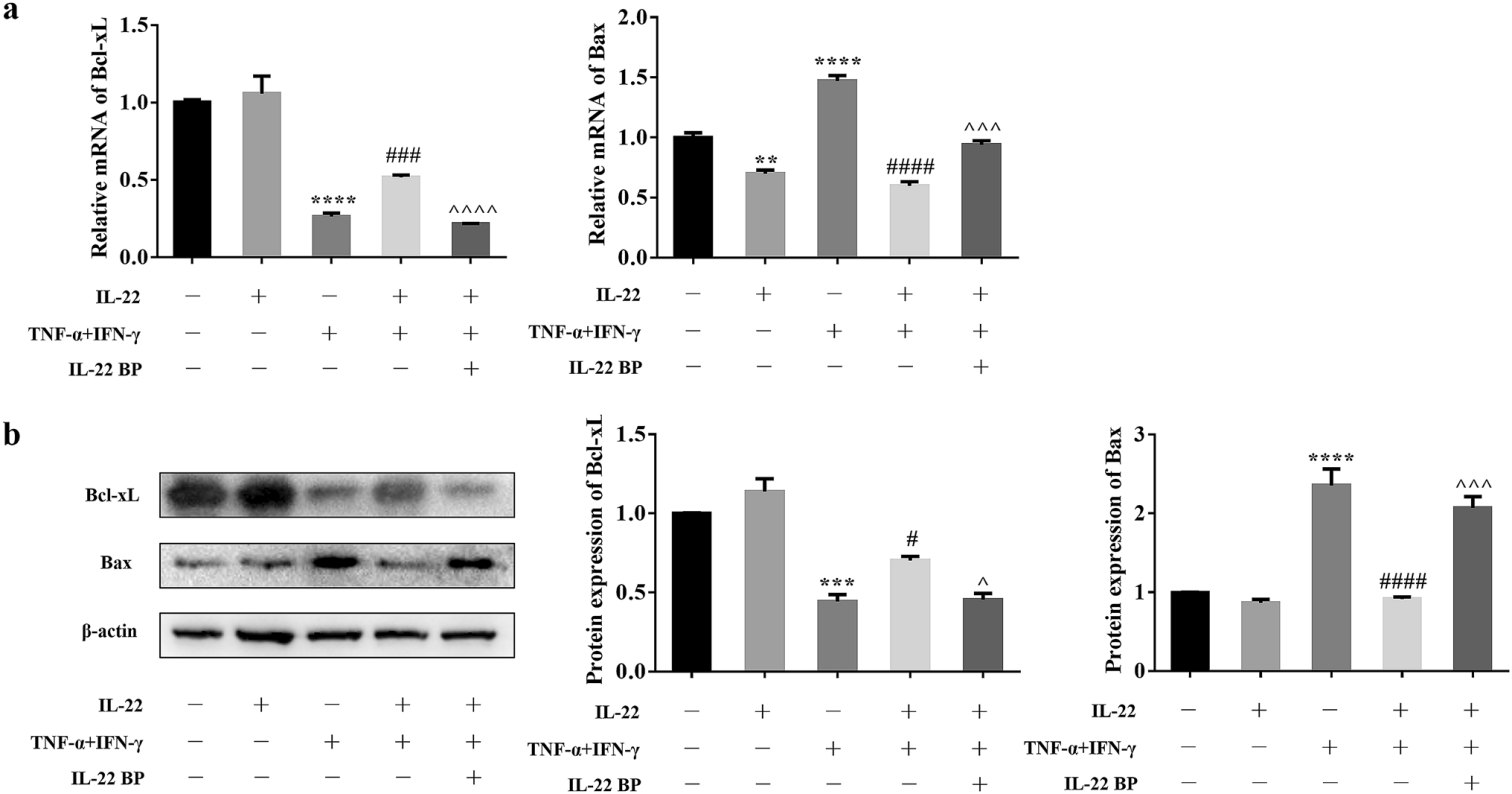
Increased Bcl-xL and decreased Bax expression in HEKs mediated with IL-22 combined with TNF-α and IFN-γ. The expression of mRNA was examined by RT-qPCR (**a**) and the expression of protein were detected by Western blotting (**b**). Statistical analysis by one-way ANOVA followed by Tukey's multiple comparisons test. (*: vs control; #: vs TNF-α + IFN-γ; ^: vs IL-22 + TNF-α + IFN-γ. **P < 0.01, ***P < 0.001, ****P < 0.0001; #*P* < 0.05, ###*P* < 0.001, ####*P* < 0.0001; ^*P* < 0.05, ^^^*P* < 0.001, ^^^^*P* < 0.0001). n ≥ 3

To further understand the mechanism of IL-22 on apoptosis, apoptosis-related genes were examined by RT-qPCR and Western blotting of HEKs treated with IL-22 after 24 h. The mRNA levels of Bcl-xL were downregulated after treatment with TNF-α and IFN-γ in HEKs by 73.74% compared with the control group (*P* < 0.0001), and IL-22 increased the expression of Bcl-xL by 96.69% compared with the group treated with TNF-α and IFN-γ (*P* < 0.001). IL-22BP completely inhibited the role of IL-22 in the presence of TNF-α and IFN-γ (*P* < 0.0001) (Fig. 5a). Similarly, the protein levels of Bcl-xL were downregulated after treatment with TNF-α and IFN-γ in HEKs by 55.64% vs. the control group (*P* < 0.001), and IL-22 increased the effect by 58.50% (*P* < 0.05). IL-22BP almost counteracted the effect of IL-22 (*P* < 0.05) (Fig. 5b).

**Fig. 6:**
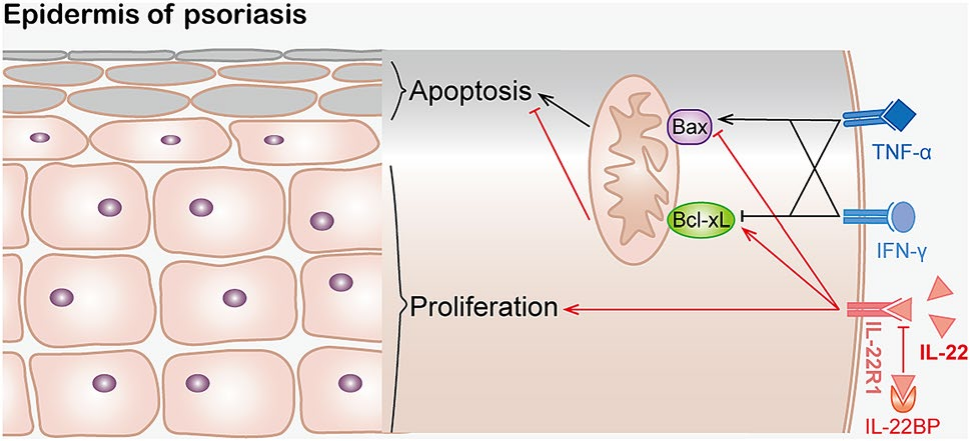
Schematic diagram depicting the mechanism of IL-22 regulating proliferation and anti-apoptosis in psoriasis. Elevated TNF-α and IFN-γ in psoriasis regulate Bax/Bcl-xL production in keratinocytes leading to apoptosis, and the increased expression of IL-22 effectively suppressed the effects through binding with IL-22R1. In addition, IL-22 can promote epidermal acanthosis alone or with other pro-inflammatory cytokines together. IL-22BP inhibits the binding of IL-22 to IL-22R1 competitively and suppresses the role of IL-22. As a result, we can observe obvious apoptotic keratinocytes in stratum corneum and abnormal hyperplasia of spinous layers

Otherwise, the mRNA levels of Bax were downregulated with IL-22 treatment of HEKs by 30.42% vs. the control group (*P* < 0.01). In contrast, the mRNA level of Bax was upregulated after treatment with TNF-α and IFN-γ in HEKs by 46.79% vs. the control group (*P* < 0.0001), and IL-22 decreased the effect by 59.39% (*P* < 0.0001). IL-22BP partly offset the role of IL-22 in the presence of TNF-α and IFN-γ (*P* < 0.001) (Fig. 5a). Likewise, the protein levels of Bax were upregulated after treatment with TNF-α and IFN-γ in HEKs by 1.36 times compared with the control group (*P* < 0.0001), and IL-22 decreased the effect by 61.23% (*P* < 0.0001). IL-22BP almost neutralized the above effect (*P* < 0.001) (Fig. 5b). Taken together, our data suggested that IL-22, regulating Bcl-xL/Bax production in HEKs in the presence of TNF-α and IFN-γ, controlled the apoptosis process, which further elucidated the anti-apoptotic mechanisms of IL-22 (Fig. 6).

## Discussion

IL-22 levels were increased in serum and lesions from psoriasis patients consistent with previous reports [4, 13, 14]. We hypothesized that the ratio of IL-22/IL-22BP could reflect the level of free bioactive IL-22. Increased IL-22/IL-22BP ratio of mRNA in psoriasis and stronger staining in epidermis further supported that the IL-22/IL-22R1 axis mainly acted on keratinocytes of psoriasis. We also found a positive correlation between the levels of IL-22, IL-22R1 and the IL-22/IL-22BP ratio and PSI. Specifically, the levels of IL-22 and IL-22R1 were elevated in those lesions with exaggerated thickness. Thus, we speculated that IL-22/IL-22R1 might be involved in the proliferation of epidermis in psoriasis. Our hypothesis was confirmed by MTT analysis. Some studies are consistent with our results, in which IL-22R1 is mainly expressed on keratinocytes, and IL-22 may induce epidermal hyperplasia by enhancing keratinocyte proliferation [20].

Apoptosis is an autonomous and orderly process of cell death [21]. Several effective treatments provoke apoptosis of proliferating keratinocyte in psoriasis patients, including methotrexate, Infliximab, natural products or herbs and narrowband ultraviolet B phototherapy [11, 22–24]. Therefore, the regression of psoriasis at least in part seems to involve apoptosis. We found that apoptotic keratinocytes were mainly observed in stratum corneum of psoriasis, almost lacking in spinous and basal layers. On the contrary, increased IL-22R1 was almost found in spinous and basal layers of psoriasis, rarely seen in stratum corneum. We speculated that the increased expression of IL-22 might participate in the proliferation of the spinous layer by inhibiting keratinocyte apoptosis, but has no effect on the apoptosis in the outermost layer. Aberrant expression of pro- and anti-apoptotic proteins in lesions may play a prominent role in the pathogenesis of psoriasis. Consistent with previous reports, the mRNA expression ratios of Bcl-xL and Bax were also elevated in the epidermis of psoriasis [25]. Furthermore, we found a positive correlation with the ratio of IL-22/IL-22BP and Bcl-xL/Bax, suggesting that IL-22 might contribute to anti-apoptosis in psoriasis.

We also observed that TNF-α and IFN-γ mRNA expression levels in lesions of psoriasis were increased, consistent with other reports [16]. TNF-α and IFN-γ are involved in inflammation and epidermal hyperplasia in psoriasis [26, 27]. Many patients have been treated effectively with TNF-α antagonists (etanercept, infliximab, and adalimumab) [28]. Until now, induction of immune cells and keratinocyte apoptosis had been postulated as one possible function of anti-TNF therapies in psoriasis [11]. Viard-Leveugle et al. reported that TNF-α and IFN-γ were potential inducers of Fas-mediated keratinocyte apoptosis in toxic epidermal necrolysis [29]. Bax protein was upregulated in primary endothelial cells stimulated with TNF-α alone or in combination with IFN-γ to induce apoptosis [30]. Additionally, IFN-γ and TNF-α induced apoptosis of intestinal epithelial cells through promoting Bax expression [31], and rendered microglia sensitive to apoptosis by inhibiting Bcl-2 and Bcl-xL [32]. We also found that apoptosis of HEKs was successfully induced through up-regulation of Bax and down-regulation of Bxl-xL after treatment with TNF-α and IFN-γ. As expected, IL-22 can inhibit such apoptosis and be partially recovered by IL-22BP in HEKs. Interestingly, this regulation by IL-22 is insufficient when TNF-α and IFN-γ are lacking, because spontaneous apoptosis is rare without intervention. Therefore, the regulatory mechanism of IL-22 on anti-apoptosis of keratinocytes occurs in the presence of TNF-α and IFN-γ in psoriasis. The findings suggest that IL-22 can be involved in interaction on keratinocyte apoptosis with TNF-α and IFN-γ. Moreover, IFN-γ and TNF were able to upregulate IL‑22R1 expression in keratinocytes, which suggests that the presence of these cytokines during inflammation may amplify the actions of IL‑22 [33, 34]. As early as 2004, Radaeva et al. found stable high levels of IL-22 in HepG2 cells, constitutively activated STAT3 and induced expression of a variety of anti-apoptotic (e.g., Bcl-2, Bcl-xL, Mcl-1) proteins [35]. We also found that IL-22 upregulated the anti-apoptosis protein Bcl-xL and inhibited the pro-apoptosis protein Bax in keratinocytes. Interestingly, IL-17A alone did not regulate apoptosis on keratinocytes, the apoptotic effect of TNF-α and IFN-γ could not be influenced when we treated HEKs in vitro with a mixture of IL-17A. These findings indicate that IL-22 inhibits the apoptosis of keratinocytes independently with IL-17A. Taken together, IL-22 may play a more important role in abnormal psoriatic epidermis, rather than immunomodulation.

In conclusion, IL-22 is a key regulator of proliferation and anti-apoptosis in psoriasis through mediating the Bcl-2 family in keratinocytes and interacting with TNF-α and IFN-γ. In addition, IL-22BP can control the pathogenic actions of IL-22 in the skin as its potential therapeutic effect in epidermal alterations.

## Materials and methods

### Patients

Serum samples from 53 psoriatic patients (19–68 years, 35.79 ± 12.39, 28 males) and 30 healthy controls (23–62 years, 37.0 ± 11.72, 16 males) (age- and sex-matched) were collected for ELISA. A total of 23 plaque psoriasis patients (18–64 years, 35.70 ± 11.87, 12 males) and 12 HC (21–63 years, 37.5 ± 12.94, 7 males) (age- and sex-matched) were recruited for skin biopsy for RT-qPCR and immunohistochemistry (IHC). Skin specimens from 8 psoriasis patients (22–59 years, 36.00 ± 11.95, 5 males) and 5 HC (20–47 years, 33.40 ± 10.11, 3 males) (age- and sex-matched) were stored at -80 °C for ELISA and Western blotting. Informed consents were obtained from all subjects. All patients with psoriasis from the First Affiliated Hospital of Xi’an Jiaotong University were diagnosed according to the criteria of psoriasis. Major inclusion criteria were described as follows: no significant infections or immune suppression, and no significant hepatic, renal, or other diseases; and no systemic treatment for at least 4 weeks or topical treatment for at least 2 weeks before enrolling in the study.

### Clinical score

PSI score assessment is used to evaluate lesions of psoriatic patients before the skin biopsy. PSI-E, PSI-S and PSI-T were graded 0 to 4 (absent to very severe). The total PSI score ranges from 0 to 12 [36].

### RT-qPCR

Total RNA was isolated and purified with TRIzol reagent (Invitrogen, USA), and cDNA was synthesized from 5 μg of total RNA using a first strand cDNA synthesis kit (Fermentas, Canada) in 20 μL. RT-qPCR was performed by iQ5 (BIO-RAD, USA) with SYBR Premix Ex Taq™ II (TaKaRa, Japan) for cytokine mRNA quantitation. The following primers were used: IL-22 (NM_020525), sense primer, 5′-GCTTGACA AGTCCAACTTCCA-3′, and anti-sense primer, 5′-GCTCACTCATACTGACTCCGT-3′; IL-22R1 (NM_021258), sense primer, 5′-CTCTGCAGCACACTACCCTC-3′, and anti-sense primer, 5′-AGGAACTCTGTGTCAGGGGT-3′; IL-22BP (NM_052962), sense primer, 5′-GCCTGAACAGTCACACTTGC-3′, and anti-sense primer, 5′-GCGTTGACTGAGTTCCTGCT-3′; Bax (NM_138763), sense primer, 5′-CCCGAGAGGTCTTTTTCCGAG-3′, and anti-sense primer, 5′-CCAGCCCATGATGGTTCTGAT-3′; Bcl-xL (NM_001191), sense primer, 5′-GAGCTGGTGGTTGACTTTCTC-3′, and anti-sense primer, 5′-TCCATCTCCGATTCAGTCCCT-3′; Bcl-2 (NM_000657), sense primer, 5′-GGTGGGGTCATGTGTGTGG-3′, and anti-sense primer, 5′-CGGTTCAGGTACTCAGTCATCC-3′; TNF-α (NM_000594), sense primer, 5′-GAGGCCAAGCC CTGGTATG-3′, and anti-sense primer, 5′-CGGGCCGATTGATCTCAGC-3′; IFN-γ (NM_000619), sense primer, 5′-TCGGTAACTGACTTGAATGTCCA-3′, and anti-sense primer, 5′-TCGCTTCCCTGTTTTAGCTGC-3′; β-actin (NM_001101), sense primer, 5′-CATGTACGTTGCTATCCAGGC-3′, and anti-sense primer, 5′-CTCCTTAATGTCACGCACGAT-3′. Primers were designed and purchased (GENEWIZ, China). Gene expression was normalized by β-actin. Fold change relative to HC was calculated using the 2^−ΔΔCt^ method.

### ELISA

Human IL-22 protein expression was quantitated in patient samples using ELISA approaches according to the manufacturer’s instructions (R&D Systems, USA).

### IHC

Skin biopsies were fixed in formalin and embedded in paraffin, sectioned at 4 µm and then stained using standard approaches with rabbit polyclonal anti-IL-22R1 antibody (1:100, Abcam, UK). All positive staining from the epidermis was counted using Image-Pro Plus, version 6.0.

### TUNEL assay

TUNEL assay was used to evaluate the apoptotic response of skin tissues with paraffin-embedded using the one-step TUNEL Apoptosis Assay kit (C1088, Beyotime, China). Each tissue section was dewaxed, then added 20 μg/mL DNase free protease K, and acted at 37 °C for 30 min. After washing three times in PBS, the labeling reaction was performed using a labeling solution containing terminal deoxynucleotidyl transferase, and fluorescein dUTP at 37 °C for 60 min. Finally, tissue sections were washed in PBS, and the nuclei were stained with DAPI. The images were captured using the fluorescence microscopy (Leica DMi8, Germany). Apoptotic index was calculated as the number of apoptotic cells out of every 100 cells from five fields of each slide (× 400).

### Cytokine stimulation to HEKs

HEKs, obtained from ATCC (Manassas, VA) in 2017, were cultured in RPMI-1640 medium (HyClone, USA) containing 10% fetal bovine serum (Gibco, USA), and the cells were incubated in 5% CO_2_ at 37 °C. Briefly, after cell counting, the cell suspension was transferred to 6-well plates (Corning, USA). Nonadherent cells were removed after 24 h of culture, and RPMI-1640 medium containing 1% FBS was added. Then, recombinant human TNFα (10 ng/mL), IFN-γ (40 ng/mL), IL-22 (20 ng/mL) and IL-22BP (20 ng/mL) were added to the medium. The TNFα, IFN-γ, and IL-22 were obtained from PeproTech. The IL-22BP was obtained from R&D.

### Cell viability assay

HEKs were seeded in a 96-well plate at 3 × 10^3^ cells/well. After attachment, the cells were treated with IL-22 at 10 ng/mL, 20 ng/mL, 40 ng/mL, 80 ng/mL and 100 ng/mL or without IL-22 for 12 h, 24 h and 48 h. Then, MTT was added to the medium and incubated for 4 h at 37 °C. MTT was dissolved in DMSO for 15 min. The OD values were determined with a microtiter plate reader (Thermo, USA).

### Flow cytometry

After cytokine incubation, all cells were harvested. The cells were stained with 7AAD and Annexin V-PE Apoptosis Detection Kit (BD Biosciences, USA). The apoptosis data were analyzed by Guava flow cytometry (Millipore, USA).

### Western blotting

The protein quality was determined by Coomassie brilliant blue staining. The protein concentrations were determined with a bicinchoninic acid (BCA) reagent assay (Beyotime, China). Forty micrograms of protein were loaded into each well of a 12% gel and subjected to SDS-PAGE. Protein was transferred to 0.45 μm nitrocellulose membranes. The membranes were blocked with 5% nonfat milk in Tris-buffered saline-Tween-20 (TBST) and then incubated with rabbit monoclonal anti-Bcl-xL antibody (1:1000 dilution, Abcam, UK), anti-Bax antibody (1:1000 dilution, Proteintech, USA) and IL-22R1 antibody (1:1000 dilution, Proteintech, USA) at 4 °C overnight. The membranes were then incubated with horseradish peroxidase-conjugated anti-rabbit secondary antibody IgG (Bioss, China) for 1 h. Next, the membranes were washed 3 times with TBST and developed via enhanced chemiluminescence (ECL) plus Western blotting detection system. β-Actin (1:1000, Santa Cruz, USA) was used as the loading control. All western blotting was performed in triplicate.

### Statistical analysis

The results are expressed as the mean ± SEM. The statistical analyses were calculated using one-way or two-way ANOVA followed by Tukey's multiple comparisons test or *t* test. Correlation analysis was measured using Pearson correlation analysis. *P* < 0.05 is accepted as significant.
